# Racing Heart Under Stormy Skies: A Journey From Agranulocytosis to Thyroid Storm

**DOI:** 10.7759/cureus.106197

**Published:** 2026-03-31

**Authors:** Jia Miao Tan, Mark Vin Wong, Dorothy Maria Anthony Bernard, Siew Hui Foo

**Affiliations:** 1 Endocrine Unit, Hospital Selayang, Selayang, MYS; 2 Internal Medicine, Hospital Selayang, Selayang, MYS; 3 Endocrinology, Hospital Selayang, Selayang, MYS; 4 Medicine, Subang Jaya Medical Centre, Subang Jaya, MYS; 5 Endocrinology and Diabetes, Hospital Selayang, Selayang, MYS

**Keywords:** agranulocytosis, anti-thyroid drug, plasma exchange, thyroidectomy, thyroid storm

## Abstract

Thyroid storm is a rare, severe, and life-threatening exacerbation of thyrotoxicosis, characterized by dysfunction of the thermoregulatory, central nervous, gastrointestinal, hepatic, and cardiovascular systems. We present a 31-year-old female with newly diagnosed Graves’ disease complicated by carbimazole-induced agranulocytosis. The delay in initiating adjunctive therapy to control the thyrotoxicosis due to suspected sepsis was followed by rapid development of thyroid storm requiring therapeutic plasma exchange (TPE) and urgent thyroidectomy. This case highlights the importance of prompt recognition and of distinguishing thyroid storm from sepsis in the setting of anti-thyroid drug-induced agranulocytosis and underscores TPE as an effective, well-tolerated bridging therapy for thyroid storm before definitive treatment.

## Introduction

Thyroid storm is a severe and life-threatening exacerbation of thyrotoxicosis, characterized by multiorgan dysfunction with a mortality rate of 10-30% [1,2]. While Graves' disease is typically managed with anti-thyroid drugs (ATDs), these can cause severe adverse effects, including agranulocytosis, which is defined as an absolute neutrophil count <0.5 × 10⁹/L apart from hepatotoxicity, occurring in 0.2% to 0.5% of treated patients [3,4].

The clinical presentation of thyroid storm often mimics sepsis due to shared features such as fever, tachycardia, and multiorgan dysfunction, posing a significant diagnostic challenge, especially in patients with concomitant ATD-induced agranulocytosis at risk of infection due to severe neutropenia.

We describe a challenging case of a young woman with Graves' disease who developed carbimazole-induced agranulocytosis and subsequently thyroid storm with multiorgan failure, necessitating intensive care admission and the implementation of therapeutic plasma exchange (TPE). The timely initiation of TPE upon recognition of thyroid storm, followed by urgent thyroidectomy, contributed to her survival and recovery when the conventional treatment of ATD was contraindicated.

## Case presentation

Our patient was a 31-year-old woman recently diagnosed with hyperthyroidism and commenced on carbimazole 40 mg daily at another institution, where she presented with palpitations and diarrhea. Initial thyroid function test revealed elevated free thyroxine (FT4) of 64 pmol/L (reference range 11.9-21.6 pmol/L) and suppressed thyroid-stimulating hormone (TSH) at <0.01 uIU/mL (reference range 0.27-4.2 uIU/mL). Three weeks into treatment, she presented to the emergency department of our institution with fever, vomiting, and right hypochondriac pain. Initial vital signs showed blood pressure of 121/90 mmHg, heart rate of 92 beats/minute, respiratory rate of 18/minute, and temperature of 37 degrees Celsius. Physical examination was unremarkable with no palpable goiter. Laboratory evaluation showed hemoglobin of 9.8g/dL (reference range 12-15 g/dL), white cell count (WCC) of 4.2 × 10⁹/L (reference range 4.0-10.0 × 10⁹/L), and platelet count of 153 × 10⁹/L (reference range 150-410 × 10⁹/L). Thyroid function tests showed elevated FT4 at 31 pmol/L (reference range 7.86-14.41 pmol/L) and suppressed serum TSH at 0.03 uIU/mL (reference range 0.38-5.33 uIU/mL). There was cholestatic transaminitis with alanine transaminase (ALT) of 157 U/L (reference range 0-35 U/L), aspartate transaminase (AST) of 220 U/L (reference range 0-35 U/L), alkaline phosphatase (ALP) of 811 U/L (reference range 33-98 U/L), and total bilirubin (TB) of 44.3 µmol/L (reference range 5-21 µmol/L). The laboratory abnormalities are summarized in Table 1.

**Table 1:** Laboratory investigation results and timeline of key clinical events ALP, alkaline phosphatase; ALT, alanine transaminase; ANC, absolute neutrophil count; APTT, activated partial thromboplastin time; AST, aspartate transaminase; CBZ, carbimazole; FFP, fresh frozen plasma; FT4, free thyroxine; G-CSF, granulocyte colony-stimulating factor; INR, international normalized ratio; N/A, not available; N/D, not done; NR, normal range; PT, prothrombin time; TB, total bilirubin; TPE, therapeutic plasma exchange; TSH, thyroid-stimulating hormone; WCC, white blood cell count

	18.8.24	20.8.24	22.8.24	23.8.24	24.8.24 -25.8.24	26.8.24	27.8.24	28.8.24
	Day 1	Day 3	Day 5	Day 6	Day 7-8	Day 9	Day 10	Day 11
TSH (NR 0.38-5.33 uIU/mL)	0.03	N/D	N/D	N/D	N/D	0.03	0.01	N/D
									FT4 (NR 7.86-14.41 pmol/L)	31	N/D	N/D	N/D	N/D	44.5	45.7	N/D
ANC (NR 2.0-7.0 × 10⁹/L)	N/A	N/D	0.11	0.18	0.68 to 4.03	7.07	N/D	1.88									
WCC (NR 4.0-10.0 × 10⁹/L)	4.2	N/D	2.28	2.73	3.59 to 6.55	8.51	N/D	3.34
									PT	13.1	N/D	13.9	14.3	N/D	N/D	30.3	15.9
APTT	46.9	N/D	39.6	36.1	N/D	N/D	158	63.4
INR	0.96	N/D	1.03	1.06	N/D	N/D	2.38	1.2
TB (NR 5-21 μmol/L)	44.3	32.4	21	25.8	31.6	65	129.4	134
									ALT (NR 0-35 U/L)	157	140	86	55.4	49.8	169	722	329
AST (NR 0-35U/L)	220	189	90	51	31.6	663	4172	2353
ALP (NR 33-98 U/L)	811	693	520	432	425	526	521	306
Clinical event	Afebrile	Low-grade fever	First spike of fever to 38.5℃	Persistent spiking temperature	Spiking temperature to 40.8℃ with persistent vomiting, hypotension and tachycardia	Hypotensive, tachycardic, clinically lethargic	Pulmonary congestion	Defervescence of fever, hemodynamic parameters stabilized
						Thyroid storm diagnosed	Worsening liver failure and hypotension	-
Treatment	Hospital admission	CBZ withheld	G-CSF initiated	-	Procalcitonin 0.27 ng/mL	Transfer ICU	First cycle TPE (2.5L FFP) administered	-
	-	-	-	-	G-CSF stopped	Awaiting TPE	-	-
	-	-	-	-	-	IV hydrocortisone 100 mg 8 hourly	-	-
	-	-	-	-	-	IVI esmolol infusion	-	-

**Figure 1:**
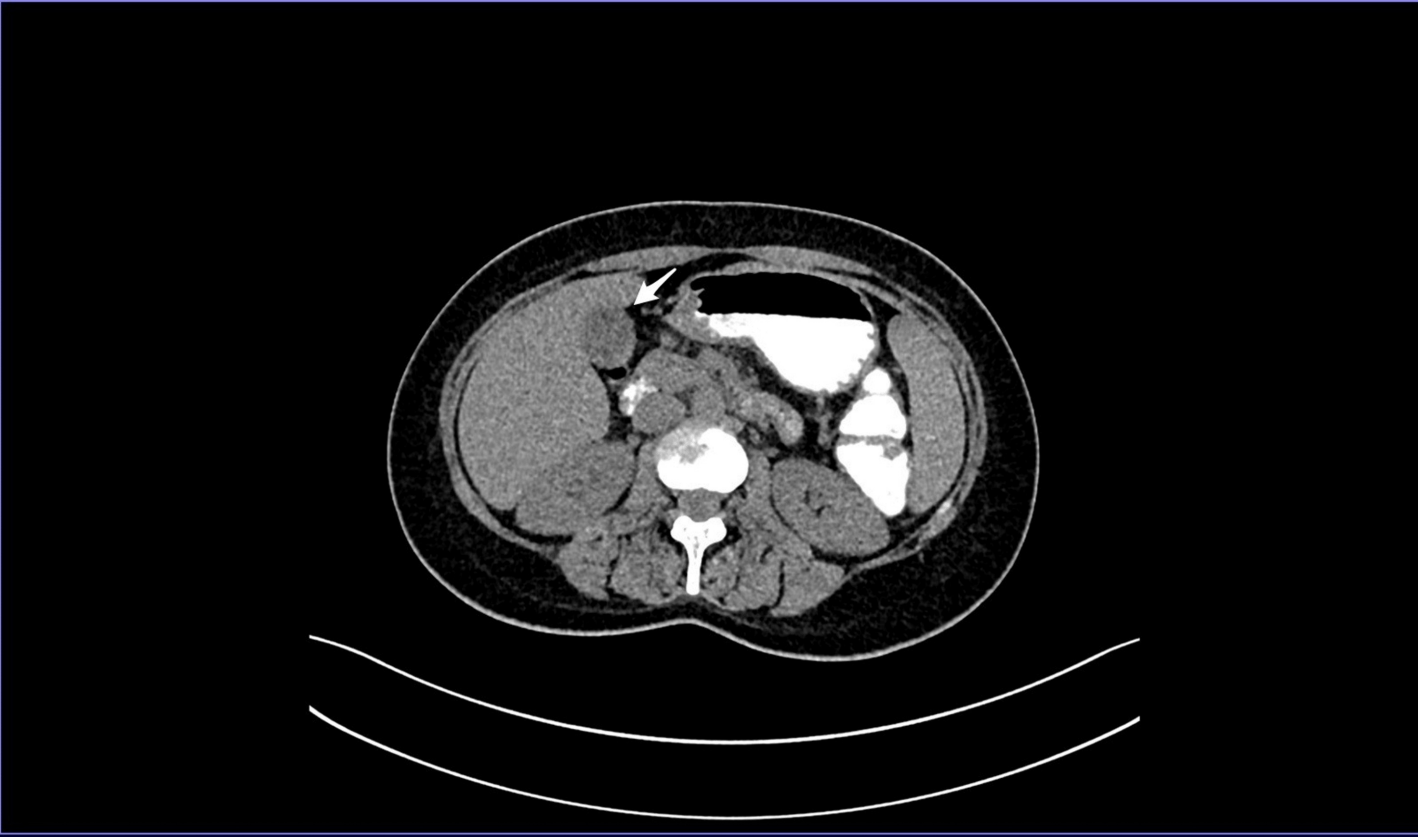
Computed tomography (CT) of the abdomen Contrast-enhanced axial CT abdomen demonstrating gallbladder wall thickening and pericholecystic inflammatory changes (white arrow) without evidence of gallstones, suggestive of acute acalculous cholecystitis.

Computed tomography (CT) of the thorax, abdomen, and pelvis performed two weeks earlier for transaminitis from another institution was reported as acute acalculous cholecystitis with no evidence of biliary dilatation (Figure 1).

Thyrotropin receptor antibody (TRAB) sampled after three weeks of carbimazole was elevated at 7.05 IU/L (reference range <1.75 IU/L), confirming the diagnosis of Graves’ disease.

**Figure 2:**
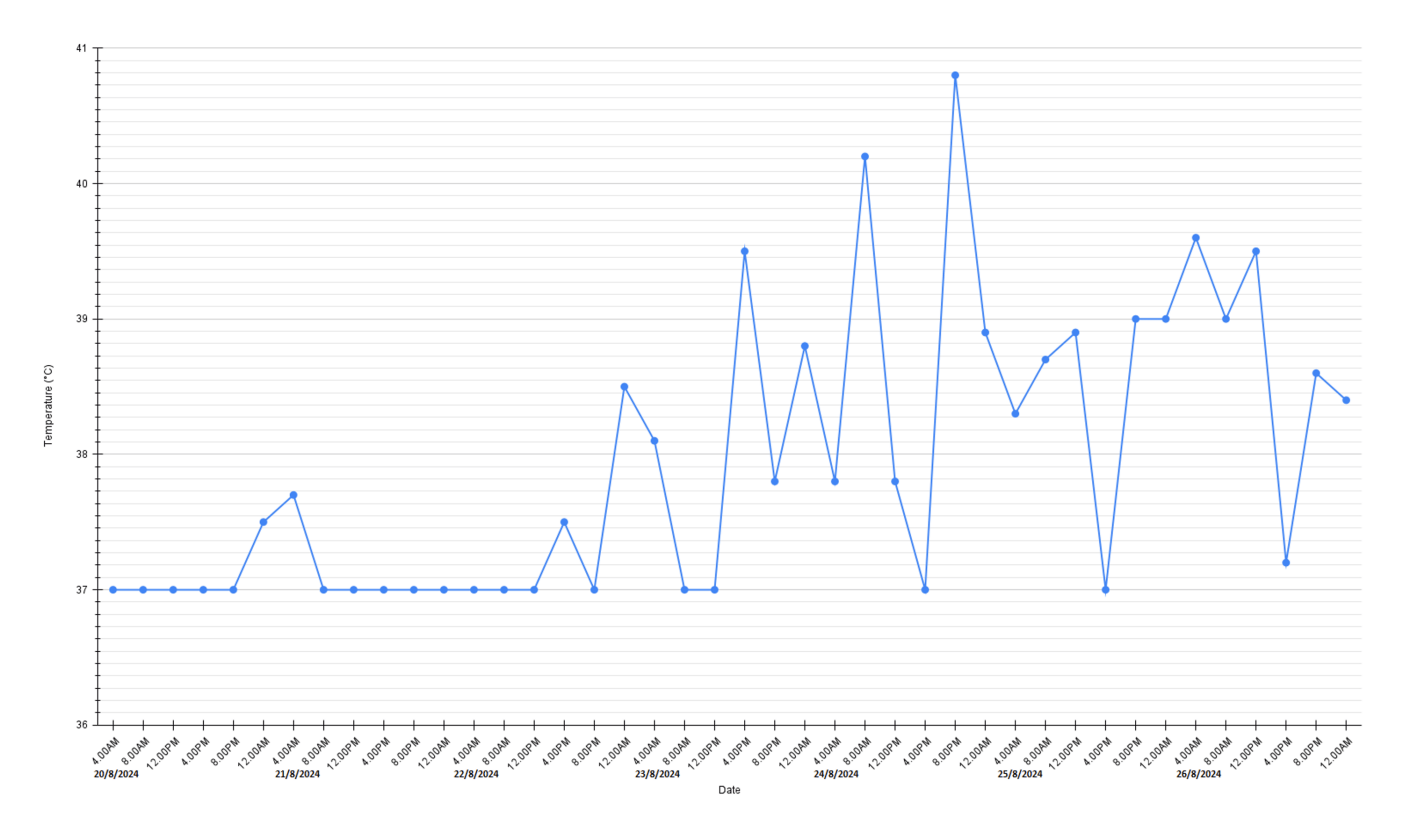
Temperature chart 20.8.2024-26.08.2024 (day 3 to day 9 admission) Image was created using Google Sheets (Google, Inc., Mountain View, CA).

On day 3 of admission, the WCC dropped to 2.38 × 10⁹/L with a markedly reduced absolute neutrophil count (ANC) of 0.11 × 10⁹/L associated with low-grade fever. A diagnosis of carbimazole-induced agranulocytosis was made, and carbimazole was withheld. Granulocyte colony-stimulating factor (G-CSF) was initiated along with a broad-spectrum antibiotic to cover for neutropenic sepsis. The WCC and neutrophil count normalized after three days of G-CSF, but she developed worsening high-grade fever of up to 40.8 degrees Celsius associated with persistent vomiting, followed by worsening hypotension and tachycardia while on broad-spectrum antibiotics. The temperature trend is illustrated in Figure 2.

Initiation of glucocorticoids, planned upon withholding of carbimazole earlier, was delayed due to concern of sepsis. Biochemistry revealed worsening FT4 to 44.5 pmol/L with suppressed TSH, along with worsening cholestatic transaminitis and rising TB with predominant conjugated hyperbilirubinemia (Table 1). Diagnosis of thyroid storm was finally made on day 9 of admission with a Burch-Wartofsky score of 60 points based on the rapid development of multiple organ decompensation with uncontrolled thyrotoxicosis despite resolution of agranulocytosis on broad-spectrum antibiotic, along with negative cultures and low procalcitonin level of only 0.27 ng/mL (Table 2).

**Table 2:** The BWPS criteria BWPS scoring in this patient supports the diagnosis of thyroid storm. bpm, beats per minute; BWPS, Burch-Wartofsky Point Scale

BWPS domain	BWPS category (criteria)	Patient finding	Scoring points
Thermoregulatory dysfunction (temperature)	39.4-39.9℃	39.5℃	25
Cardiovascular dysfunction (heart rate)	90-109 bpm	108 bpm	5
Congestive heart failure	Moderate (bibasilar rales)	Bibasal lung crepitations	10
Gastrointestinal/hepatic dysfunction	Moderate (nausea/vomiting)	Vomiting	10
Central nervous system disturbance	Absent	Absent	0
Precipitating event	Positive	Yes	10
Total score			60

**Figure 3:**
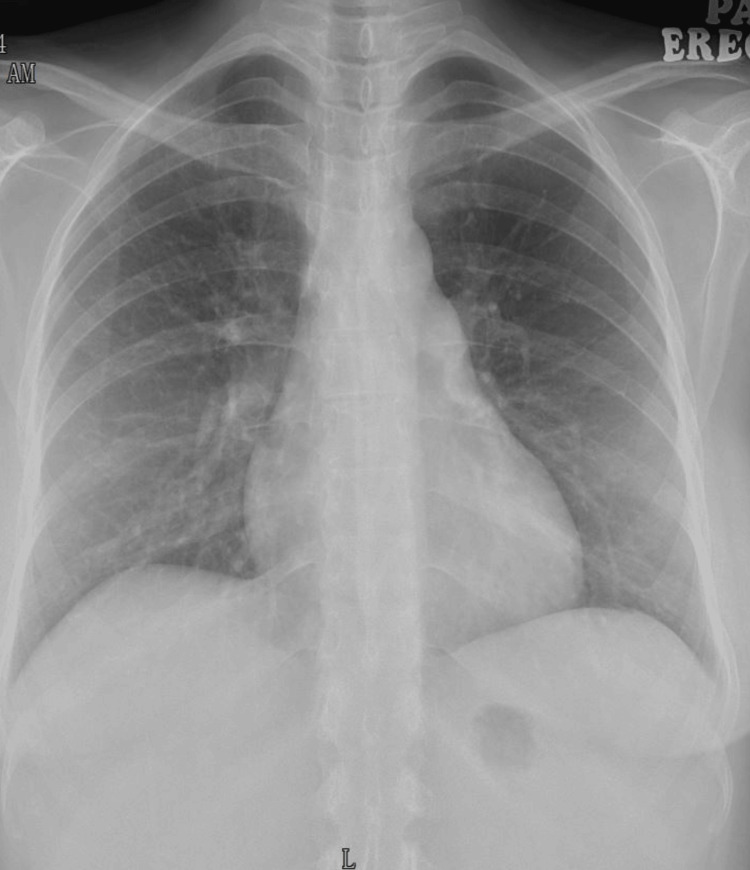
Chest X-ray on admission Chest radiograph on admission showing clear lung fields.

**Figure 4:**
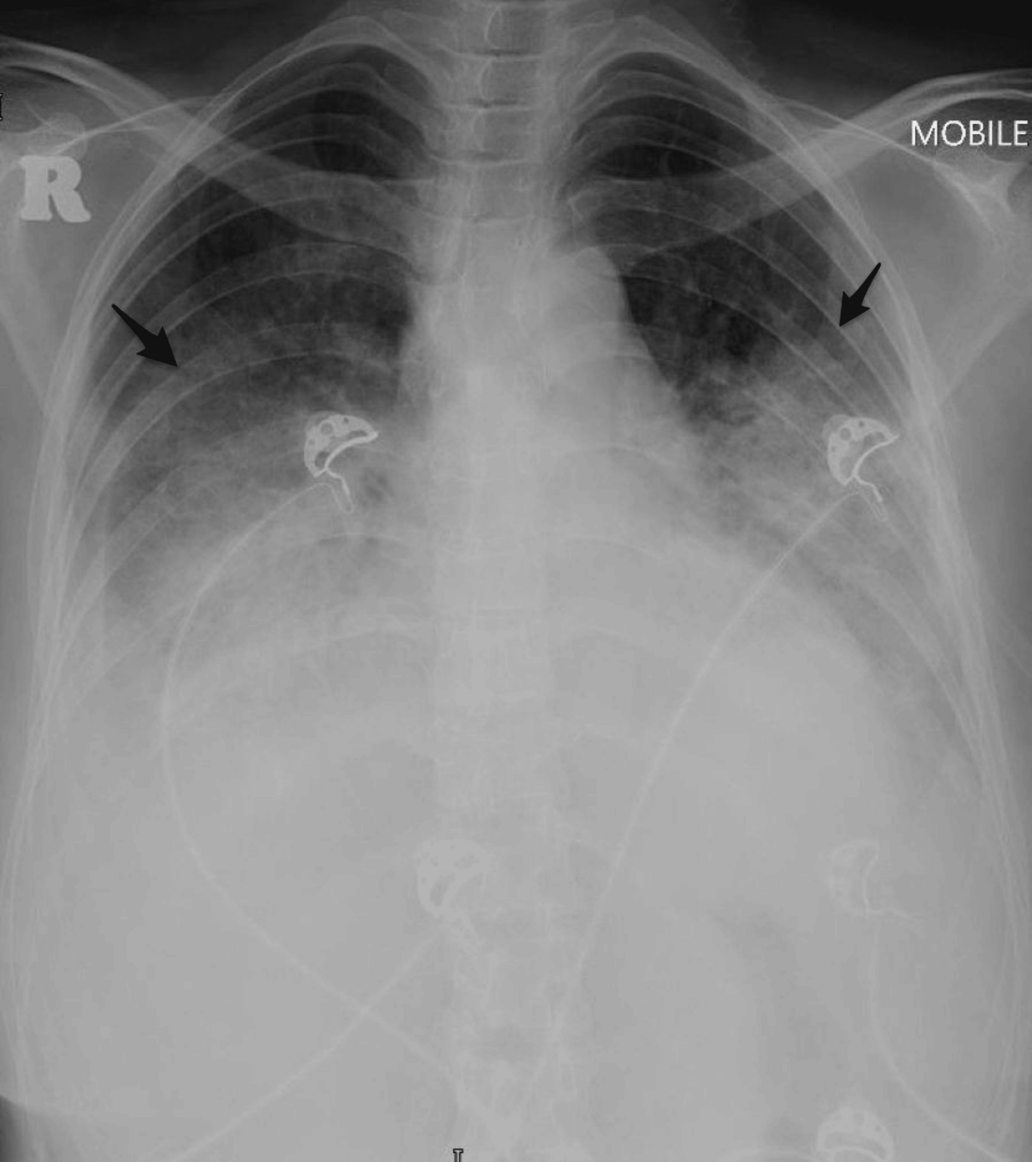
Chest X-ray on 27.8.2024 (day 10 of admission) Repeated chest radiograph at day 10 of admission showing bilateral interstitial-alveolar opacities in the perihilar regions (black arrows), in keeping with pulmonary congestion.

Urgent TPE was decided, followed by thyroidectomy, before further clinical deterioration due to thyroid storm, as high-dose ATD could not be administered due to carbimazole-induced agranulocytosis. She was transferred to the intensive care unit and started on intravenous (IV) hydrocortisone and IV esmolol infusion while awaiting TPE. She deteriorated further over the next 12 hours with type 1 respiratory failure due to pulmonary congestion requiring high flow nasal cannula and liver failure with coagulopathy. Serial chest radiographs showed worsening of pulmonary congestion (Figures 3-4).

Repeated ultrasound of the abdomen only showed minimal ascites with no evidence of biliary obstruction.

**Figure 5:**
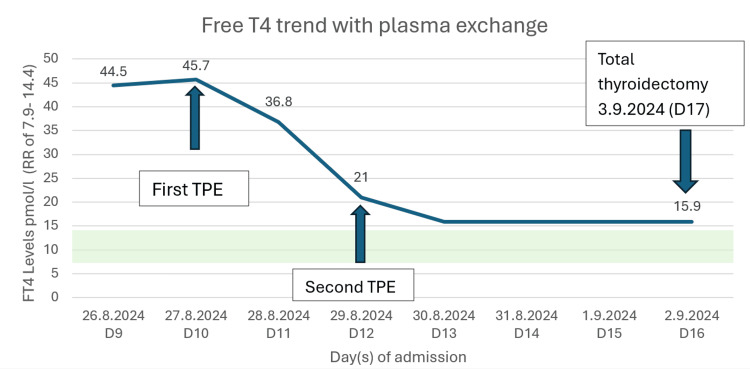
Evolution of free thyroxine serum concentrations before and after therapeutic plasma exchange FT4, free thyroxine; RR, reference range; TPE, therapeutic plasma exchange Image was created using Microsoft Word (Microsoft® Corp., Redmond, WA).

The first cycle of TPE performed on day 10 was followed by immediate clinical improvement with defervescence of fever and hemodynamic stabilization. The FT4 level improved to 36.8 pmol/L and then 21 pmol/L after the second cycle of TPE with 3 L of fresh frozen plasma (FFP) on day 12 (Figure 5).

She continued to improve clinically with resolution of coagulopathy and gradual improvement of the other liver parameters. She developed transient mild to moderate neutropenia, with an ANC of 0.7-1.1 × 10⁹/L between the two cycles of TPE, which resolved on its own over three days. Total thyroidectomy was performed after two cycles of TPE as FT4 became near normal. She was finally discharged well on day 30 after recovery from a bout of line-related sepsis.

## Discussion

ATD-induced agranulocytosis is a rare but potentially life-threatening adverse effect of commonly prescribed ATDs [3]. The reported incidence is 0.2-0.5%, typically occurring within the first three months of therapy and at higher daily doses [5]. Propylthiouracil (PTU) is contraindicated in carbimazole or methimazole-induced agranulocytosis due to the risk of immunological cross-reactivity [4]. In most cases, recovery of agranulocytosis can be expedited with G-CSFs and broad-spectrum antibiotics to cover for neutropenic sepsis [6].

Thyroid storm is a severe life-threatening exacerbation of thyrotoxicosis, characterized by multiorgan dysfunction with a high mortality rate of 10-30% [1,2]. Standard first-line pharmacotherapy in thyroid storm aims to block the production and release of thyroid hormones with high-dose ATDs, inhibit the release of pre-formed thyroid hormones with iodine, corticosteroids to inhibit peripheral conversion of T4 to T3, and control adrenergic overactivity with beta-blockade before definitive treatment in the form of radioactive iodine (RAI) ablation or surgery [1].

Hepatic dysfunction in severe thyrotoxicosis and thyroid storm is often multifactorial. In our patient, the initial transaminitis, conjugated hyperbilirubinemia, and elevated ALP likely reflected direct thyrotoxic liver injury, characterized by increased hepatic metabolic activity and oxygen consumption, leading to relative hepatocyte hypoxia and oxidative stress from free-radical generation. Furthermore, triiodothyronine (T3) has been shown to induce hepatocyte apoptosis through mitochondria-dependent pathways [2]. Carbimazole-induced hepatotoxicity, an idiosyncratic reaction typically occurring within the first 120 days of therapy, could not be entirely excluded, as it may present with a similar cholestatic biochemical pattern [3]. As the patient’s condition progressed to thyroid storm, the development of high-output cardiac failure with pulmonary congestion further contributed to congestive hepatopathy. The subsequent marked elevation in transaminases and bilirubin was consistent with ischemic hepatitis resulting from the combined effects of hepatic venous congestion, systemic hypotension, and the increased metabolic demands during the thyrotoxic crisis.

The occurrence of ATD-induced agranulocytosis and thyroid storm within the same clinical setting is infrequently reported, with fewer than five documented cases [6-9]. The challenge lies firstly in differentiating thyroid storm from neutropenic sepsis due to overlapping clinical features as reported by Rayner et al. [6]. Although the Burch-Wartofsky score is a sensitive diagnostic tool, it relies on clinical judgment and lacks specificity in the setting of suspected sepsis. It is crucial to recognize that in severe thyrotoxicosis, fever is not invariably of infectious origin; instead, it can directly result from a marked increase in the basal metabolic rate and uncontrolled thermogenesis induced by excess thyroid hormones. Our patient’s presentation of thyroid storm was initially managed as sepsis associated with persistent fever, tachycardia, and gastrointestinal symptoms that are common in both conditions during the recovery of carbimazole-induced agranulocytosis with G-CSF. Her progressive multi-organ dysfunction and worsening high-grade fever with uncontrolled thyrotoxicosis despite resolution of neutropenia with broad-spectrum antibiotic and G-CSF strongly argued against an isolated infectious process. This was further supported by negative cultures and low procalcitonin, which carries a high negative predictive value for bacterial sepsis. Her rapid defervescence and profound clinical improvement observed following TPE provided compelling evidence that thyroid storm, rather than infection, was the primary driver responsible for the multiorgan decompensation.

The second challenge in managing our patient was due to the contraindication of high-dose ATD and iodine, the conventional treatment modalities for thyroid storm, and the rapid development of multi-organ failure within hours. PTU was contraindicated as it carries the risk of cross-reactivity with carbimazole, along with hepatotoxicity [3]. Adjunctive therapies, such as cholestyramine and lithium, were deemed too slow-acting with limited potency to be able to reduce the thyroid hormone levels in time to arrest the rapid clinical deterioration due to thyroid storm [10]. Lithium was also associated with a narrow therapeutic window, nephrotoxicity, and unpredictable kinetics in the setting of acute hepatic dysfunction and multi-organ failure [10]. Corticosteroid was initiated as an adjunctive therapy, but was not expected to be able to arrest our patient’s rapid deterioration on its own. TPE was thus considered the most effective bridging therapy for rapid biochemical control and clinical stabilization before definitive thyroidectomy.

TPE clears protein-bound substances, including the thyroxine-binding globulin (TBG), and is a recognized second-line therapy for thyroid storm with Class II indication in the 2019 American Society for Apheresis guidelines [11]. Successful use of TPE to control thyrotoxicosis in ATD-induced agranulocytosis has been reported [12-14]. However, the occurrence of agranulocytosis followed by thyroid storm is infrequently reported [6-9]. Vyas et al. reported successful treatment of thyroid storm with TPE as the bridging to thyroidectomy in the setting of ATD-induced agranulocytosis [7]. Nagarajan et al. described another case of sepsis and thyroid storm with methimazole-induced agranulocytosis where TPE was performed after limited success with adjunctive therapies [8]. In contrast, Kunz et al. successfully managed their thyroid storm patient with sepsis using adjunctive therapies alone without TPE, highlighting the variability in management approaches [9]. The use of PTU after methimazole-induced agranulocytosis had been attempted as seen in Barwinek et al., where high-dose PTU was used without recurrence of agranulocytosis but still eventually necessitated TPE, although there is potential cross-reactivity from thionamides [10,15].

Timely initiation of TPE enabled our patient to undergo thyroidectomy safely, as RAI was no longer appropriate in the setting of severe thyrotoxicosis due to its significantly slower onset of action. The FT4 levels decreased by 19.4% after the first cycle of TPE and 54% overall following two cycles, consistent with the reported reduction of 22.2 % to 88.9 % in the literature [16-18]. TPE effects are often transient and last for only 24-48 hours with potential risk of rebound thyrotoxicosis as only intravascular thyroid hormones are removed [19]. Hence, multiple cycles of TPE may be required as a temporizing measure prior to definitive treatment.

During TPE, plasma is exchanged at one to one and a half times the estimated plasma volume with FFP or albumin [12]. FFP offers a theoretical advantage over albumin as it contains TBG and transthyretin, which have a higher affinity to bind free T4 and T3 [19]. In our patient, FFP was opted for not only for its potential to bind thyroid hormones, but was also crucial to replenish clotting factors, given the patient’s significant coagulopathy due to liver failure, to mitigate bleeding risk during the subsequent thyroidectomy. TPE is generally well tolerated with only 0.6% reported severe adverse events [20]. It should be repeated every 24 hours to 72 hours until clinical improvement [9]. It is essential to offer TPE promptly in thyroid storm when conventional antithyroid treatments are contraindicated or ineffective before the development of multiple organ decompensation.

## Conclusions

This case highlights the challenges in distinguishing thyroid storm from sepsis in individuals recovering from ATD-induced agranulocytosis after withholding ATDs in the setting of uncontrolled thyrotoxicosis. Thyroid storm can closely mimic sepsis, and hence, it is crucial to maintain a high index of suspicion, as early recognition can prevent morbidity and mortality, as demonstrated in this case. TPE serves as an effective adjunctive therapy in severe thyroid storm where standard therapies are contraindicated or ineffective.
